# Up-regulation of MicroRNAs-21 and -223 in a Sprague-Dawley Rat Model of Traumatic Spinal Cord Injury

**DOI:** 10.3390/brainsci10030141

**Published:** 2020-03-02

**Authors:** Hyo-Jin Chung, Wook-Hun Chung, Sun-Hee Do, Jae-Hoon Lee, Hwi-yool Kim

**Affiliations:** 1 Department of Veterinary Surgery, College of Veterinary Medicine, Konkuk University, Seoul 05029, Korea; syouth@hanmail.net (H.-J.C.); chungwookhun@gmail.com (W.-H.C.); 2 Veterinary Clinical Pathology, College of Veterinary Medicine, Konkuk University, Seoul 05029, Korea; shdo@konkuk.ac.kr; 3 Department of Veterinary Surgery, College of Veterinary Medicine, Gyeongsang National University, Jinju 52828, Korea; jh1000@gnu.ac.kr

**Keywords:** spinal cord, spinal cord injury, microRNA, pathophysiology

## Abstract

In this experimental animal study, we examined alterations in the degree of transcription of two microRNAs (miRs)—miR-21 and -223—in a Sprague-Dawley (SD) rat model of traumatic spinal cord injury (TSCI). Depending on the volume of the balloon catheter (V), a total of 75 male SD rats were divided into the three experimental groups: the sham group (*n* = 25; V = 0 μL), the mild group (*n* = 25; V = 20 μL), and the severe group (*n* = 25; V = 50 μL). Successful induction of TSCI was confirmed on both locomotor rating scale at 4 h and 1, 3 and 7 days post-lesion and histopathologic examinations. Then, RNA isolation and quantitative polymerase chain reaction (PCR) were performed. No differences in the level of miR-21 expression were found at the first time point studied (4 h post-lesion) between the three experimental groups, whereas such differences were significant at all the other time points (*p* < 0.05). Moreover, there were significant alterations in the level of miR-223 expression at all time points studied through all the experimental groups (*p* < 0.05). Furthermore, locomotor rating scale scores had a linear relationship with the level of miR-21 expression (R^2^ = 0.4363, Y = 1.661X + 3.096) and that of miR-223 one (R^2^ = 0.9104, Y = 0.8385X + 2.328). Taken together, we conclude that up-regulation of miR-21 and -223 might be closely associated with progression and the early course of TSCI, respectively.

## 1. Introduction

Traumatic spinal cord injury (TSCI) is a debilitating condition that may cause irreversible severe disability leading to motor and sensory deficits [1]. According to recent statistics, its annual incidence is estimated at 29.5 cases per million individuals on average; more than one million patients with TSCI suffer from paralysis [2]. Therefore, patients with TSCI and their caregivers are burdened with severe sequelae and high medical costs [3,4].

There is variability in the worldwide incidence of TSCI; it is estimated at 39 per million in North America, being the most prevalent; 16 per million in Australia; and 15 per million in Western Europe [5]. TSCI is considered such a serious condition that direct costs for lifetime patient care are estimated at $1.1–4.6 million per patient. It is therefore imperative that effective treatment modalities for TSCI be developed [6].

In Korea, according to the 2014 National Disability Survey, the prevalence of physical disability was estimated at 2.82% (1,373,737 patients), 4.9% (67,313 patients) of which corresponds to the percentage of TSCI (cervical, thoracic, or lumbar) and myelitis. Therefore, the prevalence of TSCI is estimated at approximately 0.1% of the total population in Korea [7].

To date, only acute methylprednisolone therapy has had protective effects on TSCI. However, its efficacy is so limited as to only marginally improve outcomes [8]. It is considered a serious health problem that may impair the quality of life in affected individuals [9]. It is therefore imperative that new therapeutic strategies be established for the treatment of patients with TSCI, for which its cellular and molecular pathophysiology should be further explored.

Pathophysiology of TSCI is composed of primary mechanical damage to the spinal cord (SC) and secondary parenchymal damage. Of the two, the latter considerably contributes to the final degree of neural damage and the severity of the long-term sequelae of TSCI [10,11,12]. The secondary parenchymal damage is of prolonged nature, based on which candidate treatment modalities have been developed [13,14,15,16]. The severity of the initial TSCI may serve as an indicator of the characteristics and degree of the secondary response; it may also be used to determine optimal treatment modalities [17,18].

Biomarkers associated with the severity of TSCI should play a role in not only assessing biological effects of a candidate treatment modality but also locating potential treatment targets [19].

According to experimental studies, miRNA (miR)-21 and -223 are biomarkers that are involved in the apoptosis and the acute phase of inflammation, respectively, both of which are features of TSCI [20,21].

Given the above background, we examined the time course of miR-21 and -223 in a Sprague-Dawley (SD) rat model of TSCI.

## 2. Materials and Methods

### 2.1. Experimental Design and Setting

In the current study, we used a total of 75 male Sprague-Dawley (SD) rats (*n* = 75), aged 9 weeks, weighing 300–330 g. They were housed in the animal facility maintained at 25–28 °C with 12 h light/dark cycles and free access to water and standard rat chow.

The current experiment was approved by the Institutional Animal Care and Use Committee (IACUC) of Konkuk University, Seoul, Korea (IACUC approval #: KU12053). All the experimental procedures were performed in compliance with the revised guidelines of the US National Institutes of Health (NIH).

### 2.2. Experimental Procedures

**Figure 1:**
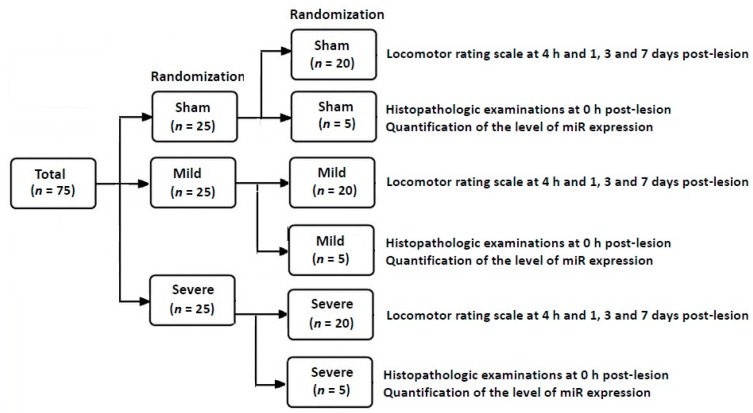
A schematic illustration of laboratory procedures.

Overall laboratory procedures are schematically illustrated in Figure 1. The SD rats were randomized to the three experimental groups after establishment of an animal model of TSCI. This is followed by confirmation of an SD model of TSCI on both locomotor rating scale scores and histopathologic findings. Then, miR samples were isolated for the quantitative real-time polymerase chain reaction (RT-PCR). Concurrently with the assessment of locomotor rating scale scores, the level of miR expression was quantified.

#### 2.2.1. Establishment of an SD Model of TSCI

We established an SD model of TSCI using a balloon-compression technique, which has been previously described in detail [22,23,24,25]. To do this, anesthesia was induced with 3% isoflurane (Forane^®^; Choongwae Pharma, Korea) and then maintained with 2.5% isoflurane. The SD rats were placed in sternal recumbency, whose lumbosacral region was shaved prior to the treatment with povidone and alcohol. A 20-G epidural catheter (BD Perisafe^TM^; Becton Dickinson Benelux N.V., Erembodegem, Belgium) was inserted in the lumbosacral joint under the fluoroscopic guidance (Power-Mobil; Siemens Medical Solutions, Erlangen, Germany). Then, a 2-F Fogarty balloon catheter (Baxter Healthcare Corp., Deerfield, IL, USA) was inserted in the epidural space and then filled with diluted iohexol mixed with saline (1:1 ratio by volume). Following the connection of the Fogarty catheter to a 50-μL syringe, its tip was placed in the ninth thoracic vertebra (T9). This was followed by the inflation of the Fogarty catheter to final volumes of 20 and 50 μL for the mild group and the severe group, respectively, for 10 min. After the rapid deflation of the Fogarty catheter, it was removed.

For the current laboratory procedures, 75 were randomly divided into the following three experimental groups:

(1) The sham group (n = 25): The SD rats undergoing insertion of an uninflated balloon catheter
(2) The mild group (n = 25): The SD rats undergoing insertion of a 20-μL balloon catheter
(3) The severe group (n = 25): The SD rats undergoing insertion of a 50-μL balloon catheter inflated at a volume of 50 μL.

#### 2.2.2. Validation of an SD Model of TSCI

To confirm whether we successfully induced TSCI, we randomly selected a total of 60 SD rats (*n* = 60) from the three groups (20 from each experimental group) and then assessed their locomotor functions at 4 h and 1, 3, and 7 days of the onset of the TSCI, as described in a prior publication [26]. Then, the remaining 15 SD rats were sacrificed for histopathological examinations. Horizontal and transverse sections of the SC were obtained at the ninth thoracic vertebra, fixed in 10% formalin solution, embedded with paraffin, and sectioned using a microtome (Shandon AS325, Thermo Electron Corp., Waltham, MA, USA) at a thickness of 5 μm. Thus, a total of five consecutive tissue sections were collected at a 3-mm gap distance. This was followed by staining with hematoxylin and eosin (H&E, Sigma-Aldrich, St. Louis, MO, USA). Histopathologic samples were examined using a light microscope (AE31, Motic, Xiamen, China).

#### 2.2.3. Isolation of miR Samples and the RT-PCR

After histopathologic examinations, total miR samples were extracted from the parenchyma of the SC. This is followed by the quantitative RT-PCR [27,28,29,30,31,32,33].

After the extraction of total miR sample from the SC using the TRIzol reagent (Life Technologies, Carlsbad, CA, USA), its amount was accurately measured based on the ultraviolet absorbance at a wavelength of 260 and 280 nm. This is followed by gel electrophoresis. Following this, the quantitative RT-PCR was performed using the TaqMan miR assay kit (Thermo Fisher Scientific Inc., Waltham, MS, USA). The reverse transcription reaction was performed using the mature miR sample containing total miR, 50 nM stem-loop reverse transcription primer, 10× reverse transcription buffer, 100mM of each dNTP, 50 U μL MultiScribe reverse transcriptase, and 20 U μL RNase inhibitor. A 15 μL of the sample was collected and then incubated in the real-time thermal cycler (Rotor-Gene^®^ Q; Qiagen GmbH, Hilden, Germany), for which the thermal profile was as follows: 30 min at 16 °C, 30 min at 42 °C and 5 min at 85 °C.

For the quantitative RT-PCR, we used the Rotor-Gene^®^ Q (Qiagen GmbH, Hilden, Germany, 72-well rotor). The reaction was performed in a final volume of with a 10 μL containing 1.33 μL of the reverse transcription product, 5 μL of 2×TaqMan Universal PCR Master Mix, 0.2 μM TaqMan probe, 15 μM forward primer, and 0.7 μM reverse primer. The thermal profile for the RT-PCR was as described: 10 min at 95 °C, then 40 cycles of 15 s at 95 °C and 1 min at 60 °C. Any discrepancies in the amount of miR between the miR samples were resolved using the U6 serving as a control. The threshold cycle (C_t_) was examined in the exponential phase of RT-PCR amplification. Then, the relative level of miR expression was analyzed using standard curves for target genes and the endogenous control. For each miR sample, geometric means were used to calculate the ΔΔCt values, thus producing 2^−ΔΔCt^. Glyceraldehyde 3-phosphate dehydrogenase (GAPDH) served as the internal control; its ΔΔCt value was set at 1, which was used to calculate alterations in the relative level of miR expression in target genes [34,35,36,37].

### 2.3. Data Analysis

All data was expressed as mean ± SEM (SEM: standard error of the mean), and was analyzed using the Statistical Package for Social Science (SPSS) version 25.0 for windows (IBM SPSS Statistics, Armonk, NY, USA). Inter-group differences were analyzed using Duncan’s multiple range test. Moreover, a Pearson’s correlation analysis was performed to identify a significant correlation between the severity of TSCI and the level of miR-21 or -223 expression. Statistical significance was set at *p* < 0.05.

## 3. Results

### 3.1. Validation of an SD Model of TSCI

**Figure 2:**
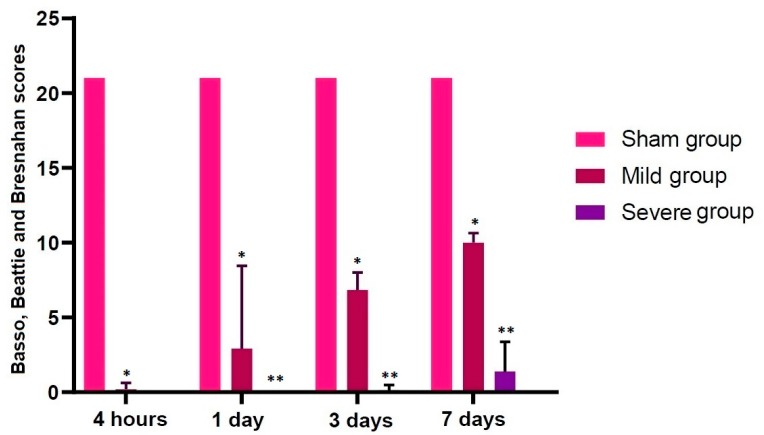
Locomotor rating scale scores. The locomotor rating scale scores remained constant in the sham group through all the time course, but the scores were drastically decreased in both the mild and severe groups when comparing with the sham group (*p* < 0.05). Asterisks indicate significant differences between the same ones at *p* < 0.05.

An SD model of TSCI was validated based on both locomotor rating scale scores and histopathological findings. As shown in Table 1 and Figure 2, the locomotor rating scale scores remained constant in the sham group through all the time course, but the scores drastically decreased in both the mild and severe groups when comparing with the sham group (*p* < 0.05).

**Figure 3:**
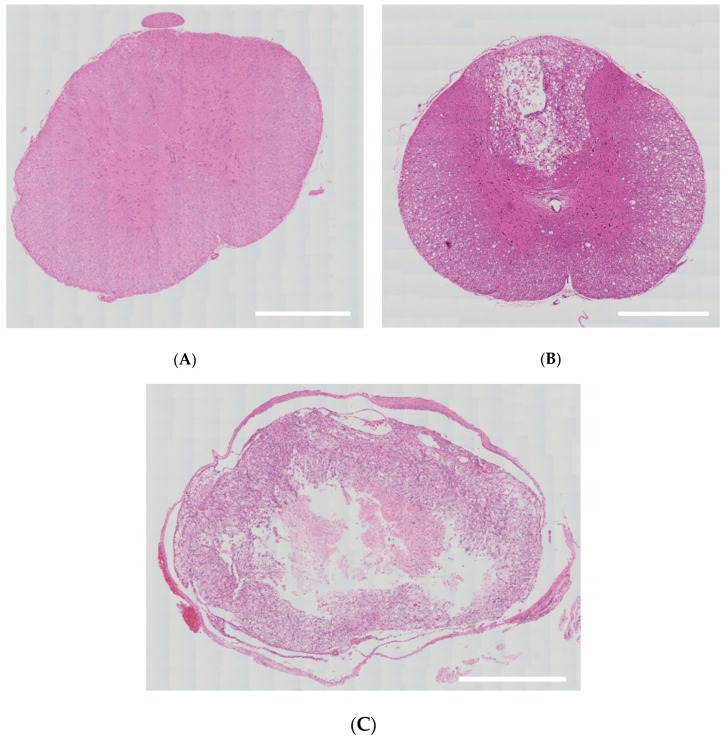
Histopathological findings. (**A**) There were no damages to the spinal cord in the sham group (36×, H&E). (**B**) There were damages to only some part of the white and gray matter in the mild group (36×, H&E). (**C**) Lesions of spinal cord were replaced by cavitation or fibrosis in the severe group (40×, H&E). Scale bar indicates 500 μm.

On histopathological findings, there were damages to only some part of the white and gray matter in the mild group. In the severe group, however, there was no remaining normal tissue in the white and gray matter; lesions of spinal cord were replaced by cavitation or fibrosis (Figure 3).

### 3.2. Alterations in the Level of miR-21 Expression according to the Time Course

**Figure 4:**
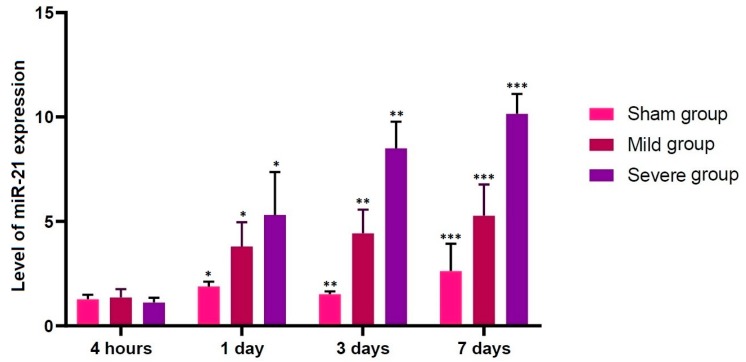
Up-regulation of miR-21. No differences in the level of miR-21 expression were found at the first time point studied (4 h post-lesion) between the three experimental groups, whereas such differences were significant at all the other time points (*p* < 0.05). Asterisks indicate significant differences between the same ones at *p* < 0.05.

No differences in the level of miR-21 expression were found at the first time point studied (4 h post-lesion) between the three experimental groups, whereas such differences were significant at all the other time points (*p* < 0.05) (Table 2; Figure 4).

### 3.3. Alterations in the Level of miR-223 Expression according to the Time Course

**Figure 5:**
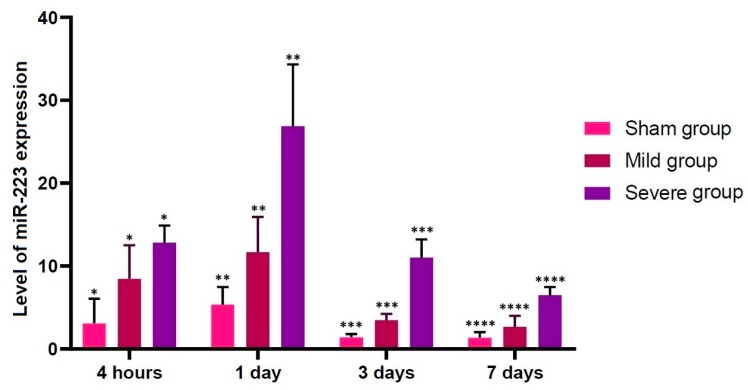
Up-regulation of miR-223. There were significant alterations in the level of miR-223 expression at all time points studied through all the experimental groups (*p* < 0.05). Asterisks indicate significant differences between the same ones at *p* < 0.05.

There were significant alterations in the level of miR-223 expression at all time points studied through all the experimental groups (*p* < 0.05). In addition, it reached the highest at 1 day but it decreased thereafter in all the experimental groups (*p* < 0.05) (Table 3; Figure 5).

### 3.4. Correlations between Locomotor Rating Scale Scores and the Level of miR-21 or -223 Expression

**Figure 6:**
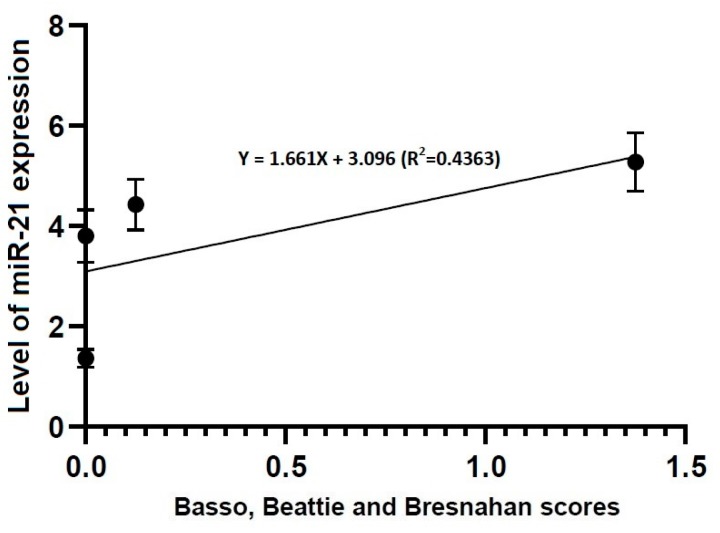
Correlation between locomotor rating scale scores and the level of miR-21 expression. The locomotor rating scale scores had a linear relationship with the level of miR-21 expression (R^2^ = 0.4363, Y = 1.661X + 3.096).

**Figure 7:**
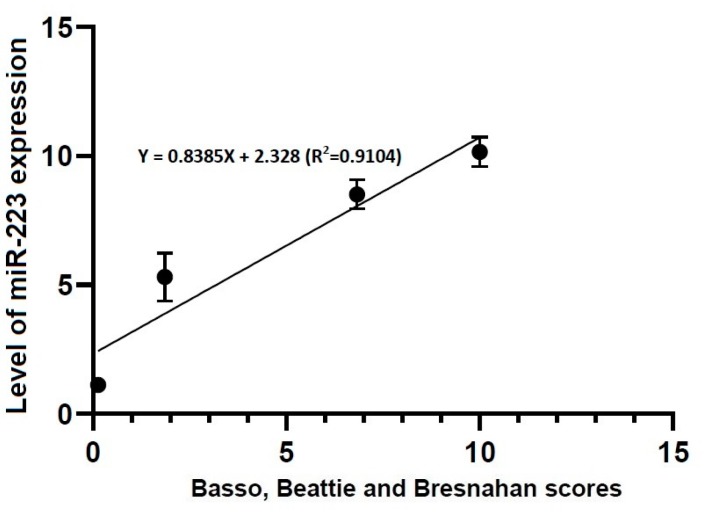
Correlation between locomotor rating scale scores and the level of miR-223 expression. The locomotor rating scale scores had a linear relationship with the level of miR-223 expression (R^2^ = 0.9104, Y = 0.8385X + 2.328).

A Pearson’s correlation analysis showed that locomotor rating scale scores had a linear relationship with the level of miR-21 expression (R^2^ = 0.4363, Y = 1.661X + 3.096) and that of miR-223 one (R^2^ = 0.9104, Y = 0.8385X + 2.328) (Figure 6 and Figure 7).

## 4. Discussion

Recent studies have reported the potential effects of miR sequences in regulating biological pathways underlying the pathophysiology of TSCI [38,39]. The miRs have a strong effect on the level of protein expression in a cellular environment. Since first identified in 1993, miR sequences have been reported to play a key role in regulating biological pathways in humans [39]. Their involvement in neurogenesis and cortical development has also been well described in the literature [40]. 

Inflammatory responses mainly constitute the secondary pathophysiology of TSCI; they are involved in regulating the pathogenesis of acute and chronic TSCI. In addition, they might play a key role in the onset of nerve injury and the control of regenerative responses [41]. Moreover, they cause apoptosis of neurons and oligodendrocytes and thereby impair neuronal functions. Furthermore, they are also involved in the formation of scar tissue [42]. This leads to the speculation that there would be a decrease in the secondary degeneration and functional deficits after the onset of TSCI if it would be possible to inhibit inflammatory responses.

Apoptosis is another feature of TSCI, and it is closely associated with nuclear DNA fragmentation and caspase activation [43]. Both neurons and oligodendrocytes undergo apoptosis in the white matter, which is accompanied by Wallerian degeneration. This greatly contributes to the paralysis of patients with TSCI [44].

In the current study, no differences in the level of miR-21 expression were found at the first time point studied (4 h post-lesion) between the three experimental groups, whereas such differences were significant at all the other time points (*p* < 0.05). We therefore assume that up-regulation of miR-21 might be closely associated with progression of TSCI. According to previous published studies, miR-21 is involved in the development and survival of neuroprogenitor cells [45]. This is in agreement with previous published studies showing that miR-21 is an antiapoptotic factor that inhibits the expression of programmed cell death protein 4 (PDCD4) and phosphatase and tensin homolog (PTEN), both of which are involved in the apoptosis [46,47].

It has been suggested that miR-21 might play a role in achieving a recovery from the TSCI. Experimental studies have shown that there was a significant increase in its expression levels at 5 weeks after the onset of TSCI in cultured astrocytes, accompanied by decreased hypertrophy due to its up-regulation and increased axonal density due to its down-regulation [48,49].

Our results showed that there were significant alterations in the level of miR-223 expression at all time points studied through all the experimental groups (*p* < 0.05). Previous published studies have shown that miR-223 is up-regulated at 4 h after the onset of TSCI [49]. Taken together, this indicates that up-regulation of miR-223 might be closely associated with early course of TSCI. According to recent studies, miR-223 is involved in the progression of TSCI; its up-regulation was observed at 12 h after the onset of TSCI on in situ hybridization (ISH) and immunohistochemistry, accompanied by over-expression of inflammatory cytokines, in an experimental model of inflammation following TSCI [21,48,50,51,52]. Furthermore, its involvement in the secondary damage to the spinal cord has also been suggested [53,54]. Thus, up-regulation of miR-223 after the onset of TSCI targets some mRNAs of anti-inflammatory cytokines [55].

To summarize, our results are as follows:

(1) No differences in the level of miR-21 expression were found at the first time point studied (4 h post-lesion) between the three experimental groups, whereas such differences were significant at all the other time points (*p* < 0.05).
(2) There were significant alterations in the level of miR-223 expression at all time points studied through all the experimental groups (*p* < 0.05).
(3) Locomotor rating scale scores had a linear relationship with the level of miR-21 expression (R^2^ = 0.4363, Y = 1.661X + 3.096) and that of miR-223 one (R^2^ = 0.9104, Y = 0.8385X + 2.328).

But our results cannot be generalized because we failed to perform miR profiling based on the ISH. As compared with other methods for detecting miRs, the ISH is more advantageous in not only monitoring their cellular and sub-cellular distributions but also determining their spatiotemporal expression profile [56,57]. In particular, the latter plays a crucial role in clarifying biological and pathologic involvement of miRs in numerous diseases [58,59]. Currently, the ISH is the only method of miR profiling that not only preserves RNA integrity but also identifies the native locations of miRs in a single cell, tissue, or cell compartments [60].

## 5. Conclusions

Although the TSCI is a serious condition that leads to debilitating outcomes, there is a limited amount of treatment resources. To date, considerable efforts have been made to clarify the pathophysiology of TSCI. This has contributed to the development of pharmacologic and cell-based therapeutic approaches, which have been accompanied by animal models showing a functional motor recovery. Of these, several therapeutics have also been tested in clinical trials [61,62].

It has been suggested that miRs are involved in the differentiation and functions of cellular components forming the spinal cord in both physiologic and pathologic conditions. The initial onset of the TSCI results in the findings that are suggestive of its secondary pathophysiology. Moreover, miRs have positive or negative effects on its onset and outcomes. That is, miRs with positive effects are involved in neuroplasticity, the regeneration of neuron and axon, re-myelination, and the recovery of normal functions [63,64,65]. It remains problematic, however, that individuals with TSCI are vulnerable to up-regulation of unfavorable miRs and down-regulation of favorable ones [66,67,68,69].

Personalized medicine for patients with TSCI would become available if biomedical researchers and clinicians could modulate miR expression levels, which would be essential for establishing novel diagnostic and therapeutic strategies for them. Hopefully, this would be followed by the validation of experimental and safety data in a clinical setting prior to the consideration of miR-based treatment strategies in humans [70,71,72,73,74].

To explain the relationship between miRs and the onset of TSCI, miRs have been categorized into hundreds of families. Therefore, identical miR families might target the same categories of genes, thus being concurrently involved in the regulation of specific physiological processes. Moreover, their potential targets also include genes involved in many pathophysiological cascades associated with the onset of TSCI, such as inflammation, apoptosis, and oxidation [51,57,75].

Based on previous experimental studies showing involvement of miR-21 and -223 in apoptosis and inflammation, respectively, constituting the secondary pathophysiology of TSCI, we have speculated that their expression levels might be of therapeutic significance [20,21,76]. We have therefore concluded that up-regulation of miR-21 and -223 might be closely associated with progression and early course of TSCI, respectively. However, this deserves further experimental studies.

## Figures and Tables

**Table 1:** Alterations in the locomotor rating scale scores according to the course.

Group	Time Points			
	4 H	1 Day	3 Days	7 Days
Sham	21.00000	21.00000	21.00000	21.00000
Mild	0.12500 *	1.85714 *	6.83333 *	10.00000 *
Severe	0.00000	0.00000 **	0.12500 **	1.37500 **

Data are mean ± SEM (SEM: standard error of the mean). Asterisks indicate significant differences between the same ones at *p* < 0.05.

**Table 2:** Alterations in the level of miR-21 expression according to the course.

Group	Time Points			
	4 H	1 Day	3 Days	7 Days
Sham	1.27396 ± 0.09624	1.88336 ± 0.10513 *	1.51919 ± 0.05863 **	2.62678 ± 0.58438 ***
Mild	1.36184 ± 0.17934	3.80291 ± 0.52056 *	4.43207 ± 0.50885 **	5.27714 ± 0.66994 ***
Severe	1.11534 ± 0.10416	5.30909 ± 0.92265 *	8.50091 ± 0.56952 **	10.1606 ± 0.42516 ***

Data are mean ± SEM (SEM: standard error of the mean). No differences in the level of miR-21 expression were found at the first time point studied (4 h post-lesion) between the three experimental groups, whereas such differences were significant at all the other time points (*p* < 0.05). Asterisks indicate significant differences between the same ones at *p* < 0.05.

**Table 3:** Alterations in the level of miR-223 expression according to the time course.

Group	Time Points			
	4 h	1 Day	3 Days	7 Days
Sham	3.068116 ± 1.348989 *	5.35168 ± 0.95415 **	1.40717 ± 0.17022 ***	1.37411 ± 0.30366 ****
Mild	8.465987 ± 1.812612 *	11.6884 ± 1.89249 **	3.47943 ± 0.33655 ***	2.67426 ± 0.59617 ****
Severe	12.8211 ± 0.921337 *	26.8762 ± 3.33326 **	11.0146 ± 0.9859 ***	6.48124 ± 0.44328 ****

Data are mean ± SEM (SEM: standard error of the mean). There were significant alterations in the level of miR-223 expression at all time points studied through all the experimental groups (*p* < 0.05). Asterisks indicate significant differences between the same ones at *p* < 0.05.
